# Effect of Sterilization on Bone Implants Based on Biodegradable Polylactide and Hydroxyapatite

**DOI:** 10.3390/ma16155389

**Published:** 2023-07-31

**Authors:** Agnieszka Kucharska-Jastrząbek, Edyta Chmal-Fudali, Daria Rudnicka, Barbara Kosińska

**Affiliations:** Institute of Security Technologies “MORATEX”, Marii Sklodowskiej-Curie 3 Street, 90-505 Lodz, Poland; ajastrzabek@moratex.eu (A.K.-J.); drudnicka@moratex.eu (D.R.); bkosinska@moratex.eu (B.K.)

**Keywords:** biodegradable implant, polylactide, hydroxyapatite, sterilization, 3D printing

## Abstract

Medical devices intended for implantation must be, in accordance with the legal provisions in force in the European Union, sterile. The effect of sterilization on the structural and thermal properties of implants, made by 3D printing from biodegradable polylactide and hydroxyapatite in a proportion of 9/1 by weight, was evaluated. The implants were sterilized using three different methods, i.e., steam sterilization, ethylene oxide sterilization, and electron beam radiation sterilization. As a result of the assessment of the structural properties of the implants after sterilization, a change in the molecular weight of the raw material of the designed implants was found after each of the performed sterilization methods, while maintaining similar characteristics of the thermal properties and functional groups present.

## 1. Introduction

Three-dimensional printing (3DP) has tremendous potential to provide rapid and customized medical models to assist in surgical planning [1] and offer great opportunities in the biomedical field, which means that, in not too distant future, 3DP may be one of the key elements for the resolution of important problems related to human health that still exist [1,2,3,4,5]. Biodegradable polymeric materials of natural or synthetic origin are usually used in 3DP implants, which favor the possibility of obtaining an ideal 3D scaffold, i.e., consisting of a biocompatible, biodegradable material with mechanical properties similar to the tissue into which it is to be implanted, which gives a chance not only for the lack of rejection or easier healing, but also allows for the possibility of earlier surgery in children (without waiting for the end of their growth period) or elimination of possible reoperation [6,7,8]. Due to the lower costs of synthetic biopolymers compared to natural biopolymers, they are gaining more widespread use, and polylactide (PLA) is one of the most commonly used biopolymers for biomaterials and tissue engineering (TE) applications [7,9,10,11,12]. Advantages of PLA as a raw material for implants, which can be produced from natural sources, mostly corn and sugar cane, include non-toxicity, biocompatibility, and biodegradability [6,7,12,13,14]. Different types of modifications and additives are used to enhance the properties of PLA-based materials, such as biodegradation rate or strength, adapted to the needs of tissue engineering, including implantable medical devices [11,12,14,15].

In research on materials based on biodegradable PLA intended for bone reconstruction applications, e.g., PLA in the form of a copolymer of L-lactide and DL-lactide, is used, with hydroxyapatite (Hap) as an additive [10,16,17,18,19]. The comonomer of L-lactide (LPLA) is the semicrystalline isomer and the comonomer DL-lactide (DLLA) is the synthetic blend of D-lactide and L-lacnaratide, and it is completely amorphous without crystalline components. The degradation time of DLPLA is between 12 and 16 months; the LPLA degradation time is much slower than that of DLPLA, requiring more than 2 years to be completely absorbed, and, as shown by Bergsma et al. [20], up to more than 5.7 years [14,21].

Human bone is a natural composite material composed largely of hydroxyapatite (60 vol%) The addition of hydroxyapatite (HAp) or nanohydroxyapatite (nHAp) into the composite material of PLA-based implants is intended to improve cell adhesion and proliferation. Hydroxyapatite is quickly absorbed into the human body and can bind to indistinguishable bone-forming unions [17,19,22].

Implants are designed to be placed inside the human body; hence, the placed devices have to be sterile. That is why the final stage of manufacturing an implantable medical device is its sterilization. It should be taken into account that the sterilization process may affect on the properties of the biodegradable implant [21,23,24]. That is why the main goal of this work was to investigate the influence of sterilization methods on the structural and thermal properties of implants made by 3D printing from biodegradable polylactide and hydroxyapatite in a proportion of 9/1 by weight. This allows the selection of the appropriate sterilization method for use in the designed 3D-printed implants from biodegradable polylactide, in the form of synthetic copolymer of L-lactide and DL-lactide in an 80/20 molar ratio, and hydroxyapatite in a proportion of 9/1 by weight, with potential use as customized biodegradable implants for bone reconstruction procedures.

## 2. Materials and Methods

### 2.1. Research Object

#### 2.1.1. Materials

The implants used in the course of this study included 19 implants in a dumbbell shape and 20 implants in a spherical sector shape, which were from the model batch. All of them were made by 3D printing on a device using Fused Deposition Modeling (FDM) technology while maintaining constant the basic parameters of the process, such as the speed of the infill/shell layer was 60/30 mm/s, the printing temperature was 190 °C, the bed temperature was 80 °C, the horizontal construction orientation, raster angle ±45°, and extrusion nozzle diameter 0.8 mm.

The composite used for the 3D printing of the implant models was a filament of bone-forming biomaterials, in which biodegradable polylactide (PLA) accounted for 90% by weight and hydroxyapatite 10% by weight. In the filament was used a synthetic copolymer of L-lactide and DL-lactide in an 80/20 molar ratio and with an inherent viscosity midpoint of 5.8 dL/g, the chemical name (3S-cis)-3,6-dimethyl-1,4-dioxane-2,5-dione, polymer with 3,6-dimethyl-1,4-dioxane-2,5-dione.

The hydroxyapatite (HAp) used in the filament also was a mineral filler for medical applications.

#### 2.1.2. Preparation Implants for Study

Samples were randomly taken from the model implants for the purpose of testing the structural and thermal properties of the implant material. The remaining implants were divided into three groups (labeled A, B, and C), comprising 5 dumbbell-shaped implants and 5 spherical sector-shaped implants, intended for sterilization by various methods.

Due to the widespread availability and low implementation costs, steam sterilization was planned, despite various assessments of its impact on PLA-based implants [21,23,24].

Apart from that, sterilization with ethylene oxide, and radiation with an electron beam, were chosen.

For the purposes of sterilization with individual methods, sterile barrier systems were selected from commercially available packaging materials, in accordance with the guidelines of the EN ISO 11607-1:2009 standard [25].

Each of the implants from groups A, B, and C was placed in an individual package in the form of a bag and enclosed on a welding machine set to operating parameters selected for the individual type of packaging.

The characteristics of the packaging, constituting the sterile barrier system and data on the sterilization process for the individual groups of implants, are presented in Table 1.

Group A implants were divided into two parts, one part of which was steam-sterilized at 121 °C for 16 min, and the second part was steam-sterilized at 134 °C for 5.5 min.

Each sterilization process was performed based on a validated method by a specialized unit listed in Table 1.

### 2.2. Organoleptic Evaluation

The sterilized implants were evaluated organoleptically in terms of their shape change due to sterilization. The organoleptic assessment was carried out visually by two observers, with reflected daylight for both sides of the implant freely placed on a flat surface in a contrasting matt color.

### 2.3. Fourier-Transform Infrared Study

The functional groups within the composite material of the implant samples were characterized using a Fourier-Transform Infrared Spectroscopy (FTIR) technique using a spectrophotometer (Thermo Scientific Nicolet iS10, Waltham, MA, USA).

In order to perform correct infrared (IR) analysis, two measurements were conducted: the spectrum of the crystal as the background, and the spectrum of the tested sample. The FTIR spectrum was determined as the ratio of the sample spectrum and the background spectrum (when performing the background spectrum, the response of the spectrometer itself is measured—without any sample). Dividing the spectrum of the sample by the background spectrum (so-called “rationing”) removes the adverse effects caused by the instrument and ambient conditions; thus, the signals present in the final spectrum come from the sample only. The tests were performed by the single-reflection method at the following settings of the device work parameters: DTGS KBr detector; measuring range: 4000 cm^−1^ ÷ 600 cm^−1^; accuracy of measurement recording: 2; mirror speed: 0.31/s; aperture: 50; minimum number of recorded scans: 32; and reflective snap of ITR type (Thermo Scientific) with the diamond crystal of the reflection angle at 45°. Each scan was repeated three times per sample and averaged.

### 2.4. Gel Permeation Chromatography

In order to determine the molecular weight and weight distribution of the composite material of the implants, gel permeation chromatography (GPC) analyses were carried out. The tests were conducted by the Department of Polymer Chemistry, Centre of Molecular and Macromolecular Studies, Polish Academy of Sciences, in Lodz, using a system that included: an Agilent Technologies 1260 Infinity pump (Agilent, Santa Clara, CA, USA), an Agilent Technologies 1100 Series autosampler (Agilent), an Optilab T-rex Wyatt refractometer (Optilab, Phoenix, AZ, USA), a DAWN Heleos-II Wyatt (18-angle) laser (Wyatt Technology, Santa Barbara, CA, USA), and 2 columns of PlGel Mixed C (Agilent). The flow rate of the mobile phase, which was methylene chloride (dichloromethane), was 0.8 mL/min. ASTRA 7.1.2 Wyatt software was used for spectrum processing.

### 2.5. Thermal Properties

The thermal analysis of the samples before and after sterilization was carried out in inert gas (nitrogen), using the differential scanning calorimeter DSC 3 Mettler Toledo, calibrated on the base of reference standards: indium and n-octane. Each thermal curve was repeated three times per sample and averaged.

The following measurement program (SEGMENTS) was used:

SEGMENT I—first heating: from 25 °C to 150 °C,

SEGMENT II—cooling: from 150 °C to 25 °C,

SEGMENT III—second heating: from 25 °C to 500 °C.

On the basis of the achieved DSC thermograms, the range of temperatures and the heating effect (enthalpy) of the crystallization and melting processes of the crystalline phase were determined. The crystallization degree of the PLA samples was calculated according to the formula presented in Equation (1):

(1) $$X_{c}=\frac{\Delta H_{m}}{\Delta H_{m}^{0}}\cdot 100\%$$

where:

*X_c_*—degree of crystallinity;
Δ*H_m_*—enthalpy connected with the melting process of the crystalline phase;
$\Delta H_{m}^{0}$—enthalpy connected with the melting process of the crystalline phase PLA; completely crystalline ($\Delta H_{m}^{0}$ = 109 J/g) [26].

### 2.6. Test of Sterility

The implant sterility tests were carried out in an accredited laboratory of SteriPack Medical Poland Sp. z o. o. using a chamber with a vertical laminar airflow, and a gas burner and incubator for microbiological cultures.

The sterility of the implants was assessed in accordance with EN ISO 11737-2:2020 [27], using the direct immersion method. Incubation was carried out for 14 days at 30 ± 2 °C with soybean casein-digested broth (TSB) as the culture medium.

## 3. Results and Discussion

### 3.1. Change in the Shape of the Implants

**Figure 1:**
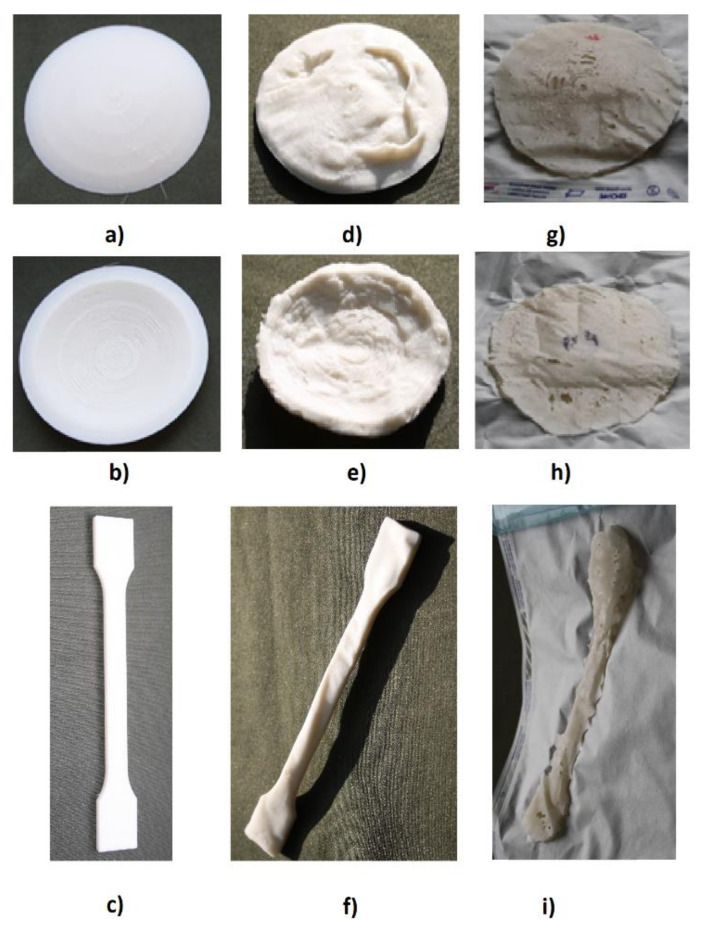
Changes in the shape of the implants due to steam sterilization. Annotation: (**a**) the top side of the implant in spherical sector shape before sterilization; (**b**) the underside of the implant in spherical sector shape before sterilization; (**c**) the paddle-shaped implant before sterilization; (**d**) the top side of the implant in spherical sector shape after steam sterilization at 121 °C for 16 min; (**e**) the underside of the implant in spherical sector shape after steam sterilization at 121 °C for 16 min; (**f**) the paddle-shaped implant after steam sterilization at 121 °C for 16 min; (**g**) the topside of the implant in spherical sector-shape after steam sterilization at 134 °C for 5.5 min; (**h**) the underside of the implant in spherical sector shape after steam sterilization at 134 °C for 5.5 min; (**i**) the paddle-shaped implant after steam sterilization at 134 °C for 5.5 min.

As a result of the organoleptic evaluation in terms of the change in the shape of the implants due to sterilization, uncontrolled deformations of the implants after steam sterilization were found, both in the process carried out at 121 °C for 16 min (Figure 1d–f) and steam-sterilized at 134 °C for 5.5 min (Figure 1g–i).

The literature mainly refers to the evaluation of sterilization in terms of the sterility of medical devices, e.g., an evaluation of the sterilization process [23,24]. We are aware that all the sterilization methods we used should ensure the sterility of the designed implants; however, the results of our research show that some of them cause unacceptable structural changes to the implants based on PLA. Changes in the shape of the implants were not observed as a result of sterilization with the ethylene oxide and radiation electron beam in the organoleptic assessment. Due to the resulting uncontrolled deformations of the implants as a result of the steam sterilization, this type of sterilization should not be used to ensure complete inactivation of microorganisms in the designed implants based on biodegradable polylactide with the addition of hydroxyapatite; therefore, sterility tests were not carried out for them.

### 3.2. The Structural Properties of the Implant Material

#### 3.2.1. Fourier-Transform Infrared Study (FTIR)

**Figure 2:**
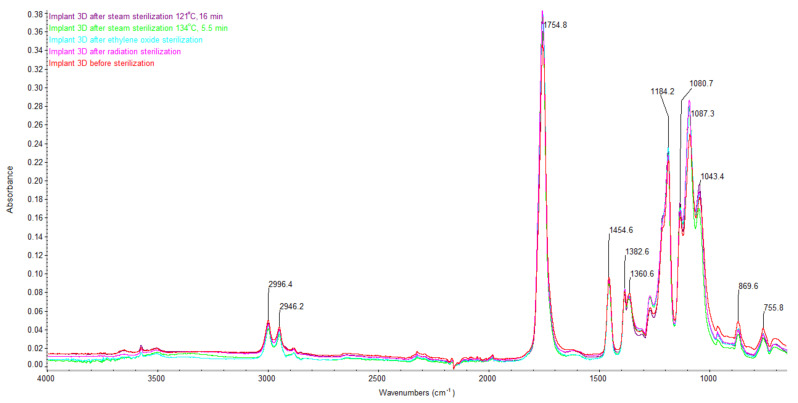
Fourier-transform infrared (FTIR) spectra of bone implant material before and after sterilization process.

The bone implant sample tested is a composite of biodegradable synthetic copolymer 80/20 (L/D,L)-PLA polylactide and hydroxyapatite. It is confirmed by the characteristic absorption bands appearing in the ATR-FTIR spectrum (Figure 2):

- ca. 3550 cm^−1^ coming from stretching vibrations of –O–H bonds;
- 2996; 2946 cm^−1^ coming from stretching vibrations of –CH– bonds;
- 1755 cm^−1^ coming from vibrations stretching the –C=O bonds;
- 1455 cm^−1^ coming from deformation vibrations of the –CH_3_ group;
- 1383; 1361 cm^−1^ originating from symmetric and asymmetric –CH– bond vibrations;
- ca. 1264 cm^−1^ coming from deformation vibrations of the –C=O bond;
- 1184; 1131; 1087 cm^−1^ coming from vibrations stretching the –C–O– bonds;
- 1043 cm^−1^ coming from vibrations stretching the –OH bonds;
- ca. 950 cm^−1^ derived from –CH in the PLA ring;
- 870 cm^−1^ coming from deformation vibrations of –C–C– bonds.

The images of the FTIR spectrum of the raw material (Figure 2) have a similar arrangement for all the examined groups of bone implants, but the sterilization process changed the intensity of the registered absorption bands. This is particularly evident for the samples after radiation sterilization and steam sterilization (Figure 2). It can be assumed that the steam sterilization process changes the PLA structure. The FTIR spectra confirmed the changes in the shape and appearance of the samples after the steam sterilization process (Figure 1g–i). The changes in the PLA structure were also confirmed by changes in the molecular weight and weight distribution of the composite material (Table 2), and also in the thermal character of the sample after the sterilization process (Table 3).

#### 3.2.2. Molecular Weight and Polydispersity

**Figure 3:**
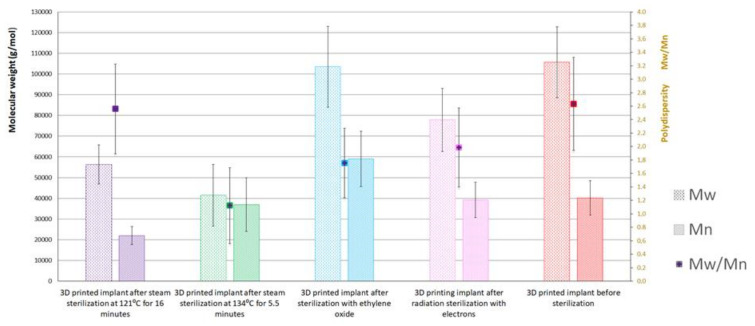
Results of gel permeation chromatography of the raw material of tested bone implants. Annotation: Mw, weight average molecular weight; Mn, number average molecular weight; Mw/Mn, polydispersity.

The results of the gel permeation chromatography (GPC) tests show that each of the sterilization methods performed changed the molecular weight of the raw material of the designed implants (Table 2 and Figure 3).

The largest changes in the weight average molecular weight (Mw) occur as a result of steam sterilization at 134 °C for 5.5 min. The Mw decreases from 10.57 × 10^4^ (±16.227%) to 4.146 × 10^4^ (±35.801%), while the number average molecular weight (Mn) changes slightly, from 4.014 × 10^4^ (±20.597%) to 3.697 × 10^4^ (±35.110%)), resulting in a very large change in polydispersity (Mw/Mn) from 2.633 (±26.221%) to 1.122 (±50.143%). The relative measurement uncertainty is also very high, about 35% for Mn and Mw, which indicative of the lack of homogeneity of the samples. The visible deformation of the implants may be a sign of uncontrolled changes in their structure.

Also, steam sterilization carried out at 121 °C for 16 min significantly changes the Mw, from 10.57 × 10^4^ (±16.227%) to 5.628 × 10^4^ (±16.850%), with the Mn being greatly reduced from 4.014 × 10^4^ (±20.597%) to 2.198 × 10^4^ (±19.830%) The relative uncertainty of measurement is still very high, but it decreases to levels similar to those obtained in the tests of the implant material before sterilization, i.e., for Mn, it is about 16%, and for Mw is about 20%. Radiation sterilization with electrons significantly reduces the Mw, from 10.57 × 10^4^ (±16.227%) to 7.789 × 10^4^ (±19.554%), while the Mn results are greatly reduced, from 4.014 × 10^4^ (±20.597%) to 2.198 × 10^4^ (±19.830%). The relative uncertainty of the measurement increases in relation to that obtained in the tests of the implant material before sterilization; for Mw, it is about 20%, and for Mn, it is about 22%.

The smallest change in Mw, from 10.57 × 10^4^ (±16.227%) to 10.35 × 10^4^ (±18.882%), is caused by ethylene oxide sterilization, with Mn increasing from 4.014 × 10^4^ (±20.597%) to 5.896 × 10^4^ (±22.611%). The relative uncertainty of measurement is at a level similar to that obtained in the study of the implant material after radiation sterilization with electrons, and slightly higher than in the study of the implant material before sterilization, i.e., for Mn, it is about 19%, and for Mw, it is about 22.6%.

Changes in the molecular weight and weight distribution of the composite material of the implants observed in the GPC studies may indicate changes in the structure of the implants that will affect their biocompatibility and biodegradability in the human body, and thus it is necessary to study implants in this area.

### 3.3. Thermal Analysis

**Figure 4:**
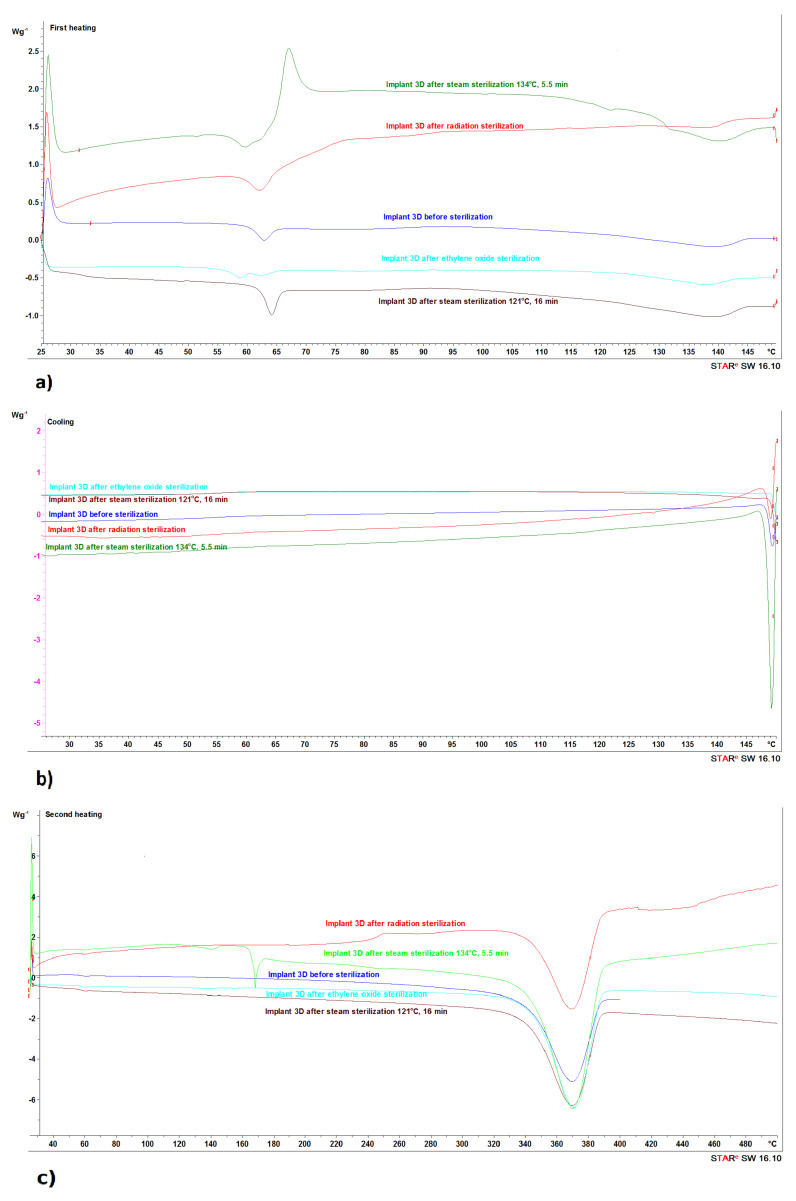
Differential scanning calorimetry (DSC) curves of bone implant material. Annotation: (**a**) first heating process 25–150 °C (SEGMENT I); (**b**) cooling process 150–25 °C (SEGMENT II); (**c**) second heating process 25–500 °C (SEGMENT III).

Differential scanning calorimetry (DSC) analysis of the tested bone implant samples was carried out in three stages (SEGMENTS). The changes taking place in the implant material, both for the unsterilized sample and after the sterilization process, for the individual stages are shown in Figure 4.

The first stage DSC study consisted in heating the sample to the temperature of 150 °C (SEGMENT I) (Figure 4a). The second stage assumed the cooling process (SEGMENT II) in order to record the reverse process related to physical transformations, which was to confirm their nature (Figure 4b). The third stage consisted in reheating the sample to 400 °C (SEGMENT III) (Figure 4c).

As a result of heating the sample of the examined bone implant before the sterilization process (SEGMENT I), two small endothermic peaks were recorded on the DSC curve (Figure 4a), whose maximum transformation temperatures were 60 °C and 137 °C (Table 3).

It was recognized that they correspond to physical (reversible) changes taking place in the structure of the tested sample. During the cooling of the sample (SEGMENT II), there are no changes in the form of peaks in the expected temperature ranges, indicating the reversible nature of the transformation (Figure 4b), but a slightly outlined step of about 55 °C, characteristic of the glass transition, was noticeable. On reheating the sample (SEGMENT III), conversions of around 56 °C and 139 °C were recorded (Figure 4c, Table 3). The first transformation recorded on the DSC curve during heating (SEGMENT I) and reheating (SEGMENT III) is related to the glass transition, while the second one is related to the melting of the crystalline phase [28]. However, taking into account that, in the case of 80/20 PLA, the crystallization process occurs only when the sample is subjected to stress (stress crystallization process [28]), no crystallization process was recorded during its cooling (SEGMENT II).

Thus, the transformation associated with the melting of the crystalline phase during repeated heating (SEGMENT III) was not registered (Figure 4c). The slight transformation recorded during the reheating (SEGMENT III) of the sample around 140 °C may, however, be due to the fact that, during the process of creating the implant with the 3D printing technique, a partial ordering in the structure of the PLA macromolecule could have occurred, resulting in a higher degree of crystallinity in the created model. This is confirmed by the recorded enthalpy value of the melting process of the crystalline phase, ΔH_m_ (Table 3). A clear endothermic peak (SEGMENT III), whose maximum transformation temperature was recorded above 350 °C, comes from a chemical transformation (irreversible) and is related to the thermal decomposition of the tested sample.

The steam sterilization process did not significantly affect the glass transition temperature; however, an increase in the melting enthalpy of the PLA crystalline phase from −8.5 J/g to −14.0 J/g (steam sterilization process at 121 °C) and −17.5 J/g (steam sterilization process at 134 °C) was observed (Table 3). An increase in the crystalline phase was also observed in SEGMENT III, recording an additional transformation related to the melting of the crystalline phase (Figure 4c, Table 3). Moreover, it was observed that as a result of heating the sample of the examined bone implant after the steam sterilization process at 134 °C (SEGMENT I), a small exothermic peak was recorded on the DSC curve around 67 °C (Figure 4a). It was observed that during the cooling of the sample (SEGMENT II) and reheating (SEGMENT III), no changes were recorded in the form of peaks in the expected temperature ranges, indicating the reversible character of the transformation (Figure 4b,c).

Identification of the additional endothermic peak recorded at 168 °C during the second heating (SEGMENT III) of the sample after steam sterilization at 134 °C will require changing the temperature range of SEGMENT I (up to 200 °C). Such research is planned for the further implementation of the project.

The sterilization process with ethylene oxide resulted in an increase in the glass transition temperature and a decrease in the crystalline phase of PLA, as evidenced by the decrease in enthalpy, ΔHm (Table 3). On the other hand, the radiation sterilization process reduced both the Tg and ΔHm values (Table 3).

### 3.4. Sterility Tests

Due to the resulting uncontrolled deformations of the implants as a result of steam sterilization (Figure 1a,b), the implants from group A (Table 1) were not evaluated for complete inactivation of microorganisms.

The sterility assessment of the implants sterilized with ethylene oxide, as well as those subjected to radiation sterilization with an electron beam, was carried out in an accredited laboratory of SteriPack Medical Poland Sp. z o. o., in accordance with EN ISO 11737-2:2020 [27], and confirmed the complete inactivation of microorganisms in the designed implants.

## 4. Conclusions

The main goal of this work was to select the appropriate sterilization method for use in the designed 3D-printed implants from biodegradable polylactide and hydroxyapatite, with potential use as customized biodegradable implants for bone reconstruction procedures.

As a result of steam sterilization, both in the process carried out at 121 °C for 16 min and steam sterilization at 134 °C for 5.5 min, uncontrolled deformation of the implants takes place, and therefore these sterilization methods should not be used for the developed composite of implants.

Sterilization with ethylene oxide, as well as radiation sterilization with an electron beam, makes it possible to ensure the sterility of the designed implants, which should be assessed in terms of their biocompatibility, biodegradation, and other properties required for implants.

Finally, taking into account the economic factor, the radiation sterilization process was selected for further work, due to its non-destructive effect on the PLA structure.

## Figures and Tables

**Table 1:** Basic properties of the sterile barrier system and the sterilization process.

Implants Group	Characteristics of the Sterile Barrier System		Characteristics of the Sterilization Process		
	Packaging	Heat Sealing Parameters	Sterilization Type	Parameters of the Sterilization Process	Sterilization Unit
A	flat paper–foil bag, TZMO_OM-191-FPPA-126	downforce: 700–850 N without the air remove T = 180 °C t = 2 s	steam	T = 121 °C t = 16 min	CitoNet-Łódź Sp. z o. o. Lodz, Poland
				T = 134 °C t = 5.5 min	
B	flat paper–foil bag, TZMO_OM-191-FPPR-033	downforce: 700–850 N without the air remove T = 180 °C t = 2 s	ethylene oxide	amount of gas used 48.2 kg, including 5.1 kg of ethylene oxide	Yavo Sp. z o.o. Belchatow, Poland
C	flat foil bag WIPAK_ ESE 1250–ESE 1250 Medi Peel	downforce: 700–850 N t_AR_ = 2 s T = 135 °C t = 0.8 s	electron beam radiation	irradiation dose 25 kGy ± 0.99% transportation speed 0.459 m/min ± 1.12%	Radiation Sterilization Plant of Medical Devices and Allografts Warsaw, Poland

Annotation: T, temperature; t, time; t_AR_, time of the air removal.

**Table 2:** Results of gel permeation chromatography of the raw material of tested bone implant.

Sample Characteristics	Molecular Weight (g/mol)		Polydispersity
	Mw	Mn	Mw/Mn
bone implant sample after steam sterilization (T = 121 °C; t = 16 min)	5.628 × 10^4^ (±16.850%)	2.198 × 10^4^ (±19.830%)	2.561 (±26.022%)
bone implant sample after steam sterilization (T = 134 °C; t = 5.5 min)	4.146 × 10^4^ (±35.801%)	3.697 × 10^4^ (±35.110%)	1.122 (±50.143%)
bone implant sample after sterilization with ethylene oxide	10.35 × 10^4^ (±18.882%)	5.896 × 10^4^ (±22.611%)	1.756 (±29.458%)
bone implant sample after radiation sterilization with electrons	7.789 × 10^4^ (±19.554%)	3.922 × 10^4^ (±22.033%)	1.986 (±29.459%)
bone implant sample before sterilization process	10.57 × 10^4^ (±16.227%)	4.014 × 10^4^ (±20.597%)	2.633 (±26.221%)

Annotation: T, temperature; t, time; Mw, weight average molecular weight; Mn, number average molecular weight.

**Table 3:** Results of thermal analysis of tested bone implant samples.

Sample Characteristics	I SEGMENT Heating			II SEGMENT Cooling		III SEGMENT Reheating		
	Tg [°C]	Tm [°C]	ΔH_m_ [J/g]	Tg [°C]	Tc [°C]	Tg [°C]	Tm [°C]	ΔH_m_ [J/g]
bone implant sample before sterilization process	60.0 ± 2.7	137.0 ± 1.8	−8.5 ± 0.7	55.0 ± 2.6	-	56.0 ± 2.7	139.5 ± 2.7	−2.2 ± 0.2
bone implant sample after steam sterilization (T = 121 °C; t = 16 min)	61.0 ± 2.8	138.0 ± 1.7	−14.0 ± 0.9	54.0 ± 1.9	-	57.0 ± 2.1	142.0 ± 2.8	−0.5 ± 0.2
bone implant sample after steam sterilization (T = 134 °C; t = 5.5 min)	56.0 ± 2.7	139.0 ± 2.3	−17.5 ± 0.7	-	-	56.0 ± 1.9	140.0 ± 2.1	−6.6 ± 0.4
bone implant sample after sterilization with ethylene oxide	56.0 ± 2.4	138.0 ± 2.1	−6.3 ± 0.8	59.0 ± 1.8	-	55.0 ± 2.4	139.0 ± 2.3	−1.3 ± 0.7
bone implant sample after radiation sterilization with electrons	63.0 ± 2.7	138.0 ± 1.7	−7.4 ± 1.1	59.0 ± 1.6	-	57.0 ± 2.2	-	-

Annotation: T, temperature; t, time; Tg, glass transition temperature; Tm, maximum melting temperature of the crystalline phase; ΔH_m_, enthalpy change related to the melting of the crystalline phase; Tc, maximum crystallization temperature.

## Data Availability

Not applicable.
